# Reliable Recurrence Algorithm for High-Order Krawtchouk Polynomials

**DOI:** 10.3390/e23091162

**Published:** 2021-09-03

**Authors:** Khaled A. AL-Utaibi, Sadiq H. Abdulhussain, Basheera M. Mahmmod, Marwah Abdulrazzaq Naser, Muntadher Alsabah, Sadiq M. Sait

**Affiliations:** 1 Department of Computer Engineering, University of Ha’il, Ha’il 682507, Saudi Arabia; 2 Department of Computer Engineering, University of Baghdad, Al-Jadriya, Baghdad 10071, Iraq; sadiqhabeeb@coeng.uobaghdad.edu.iq (S.H.A.); basheera.m@coeng.uobaghdad.edu.iq (B.M.M.); 3 Department of Architectural Engineering, University of Baghdad, Al-Jadriya, Baghdad 10071, Iraq; marwahabdalkhafaji@gmail.com; 4 Department of Electronic and Electrical Engineering, University of Sheffield, Sheffield S1 4ET, UK; mqalsabah@gmail.com; 5 Department of Computer Engineering, Interdisciplinary Research Center for Intelligent Secure Systems, Research Institute, King Fahd University of Petroleum & Minerals, Dhahran 31261, Saudi Arabia; sadiq@kfupm.edu.sa

**Keywords:** discrete Krawtchouk polynomials, Krawtchouk moments, propagation error, energy compaction, computation cost

## Abstract

Krawtchouk polynomials (KPs) and their moments are promising techniques for applications of information theory, coding theory, and signal processing. This is due to the special capabilities of KPs in feature extraction and classification processes. The main challenge in existing KPs recurrence algorithms is that of numerical errors, which occur during the computation of the coefficients in large polynomial sizes, particularly when the KP parameter (*p*) values deviate away from 0.5 to 0 and 1. To this end, this paper proposes a new recurrence relation in order to compute the coefficients of KPs in high orders. In particular, this paper discusses the development of a new algorithm and presents a new mathematical model for computing the initial value of the KP parameter. In addition, a new diagonal recurrence relation is introduced and used in the proposed algorithm. The diagonal recurrence algorithm was derived from the existing *n* direction and *x* direction recurrence algorithms. The diagonal and existing recurrence algorithms were subsequently exploited to compute the KP coefficients. First, the KP coefficients were computed for one partition after dividing the KP plane into four. To compute the KP coefficients in the other partitions, the symmetry relations were exploited. The performance evaluation of the proposed recurrence algorithm was determined through different comparisons which were carried out in state-of-the-art works in terms of reconstruction error, polynomial size, and computation cost. The obtained results indicate that the proposed algorithm is reliable and computes lesser coefficients when compared to the existing algorithms across wide ranges of parameter values of *p* and polynomial sizes *N*. The results also show that the improvement ratio of the computed coefficients ranges from 18.64% to 81.55% in comparison to the existing algorithms. Besides this, the proposed algorithm can generate polynomials of an order ∼8.5 times larger than those generated using state-of-the-art algorithms.

## 1. Introduction

Digital image processing plays an essential role in several aspects of our daily lives. Image signals are subject to several processes such as transmission [1], enhancement [2], transformation [3], hiding [4], and compression [5,6]. Similarly to image processing, speech signals processing is also essential [7], and involves several stages such as transfer [8], acquisition [9], and coding [10]. Pattern recognition, which is considered an automated process, is widely used in various applications such as computer vision [11], statistical data analysis [12], information retrieval [13], shot boundary detection [14], and bio-informatics [15]. However, the accuracy of extracting the significant features in these essential signal processing approaches is crucial [16]. Feature extraction, in particular, is used to reduce the dimensionality of the signals to a finite size [17,18]. Specifically, a finite number of features can be used to represent the signals. These finite features can be considered the most significant ones and need to be extracted using efficient methods. As such, to achieve the best signal representation, a fast and robust feature extraction mechanism becomes necessary. To this end, such features’ extraction mechanism needs to meet the desired accuracy concerns by extracting the most significant features efficiently with low processing times. Furthermore, the energy compaction and localization of the signals can also be considered as essential factors in signal compression [19]. This is attributed to the fact that using fewer effective coefficients results in a more accurate representation of the signals. Hence, orthogonal polynomials are an effective tool that can be applied to meet these desired requirements and features characterization.

Continuous and discrete orthogonal polynomials are commonly used in many signal processing applications and feature characteristics. Continuous orthogonal polynomials are used in speech and image applications, for example, in pattern recognition, robot vision, face recognition, object classification, hiding information, data compression, template matching, and in edge detection for image data compression [20,21,22,23]. The performance of orthogonal polynomials is evaluated according to their ability to extract distinct features from signals in a fast and efficient way. This special ability of feature extraction can be quantified using properties such as (a) energy compaction; (b) signal representation without redundancy; (c) numerical stability; and (d) localization [24,25,26].

Discrete orthogonal polynomials are widely used to extract features from images [27]. There are different types of discrete polynomials. Examples of these include discrete Tchebichef polynomials [28], Chebyshev polynomials [29], discrete Charlier polynomials [26], discrete Krawtchouk polynomials (DKPs), and discrete Meixner polynomials [30]. Among these polynomials, DKPs are widely exploited in image processing. This is due to their salient characteristics, which can be used to extract local features from images. Specifically, by exploiting the localization property of the DKPs, images can be efficiently represented by using a finite number of features [31]. The localization property is carried out by controlling the parameter value (*p*) of the DKPs. Typically, discrete orthogonal moments are generated using DKPs. Discrete orthogonal moments are extensively exploited in image and signal processing [11,32,33,34], coding theory [35], and information theory [36,37]. However, reconstructing a signal using moments and maintaining the orthogonality has to date been considered a challenging task. In addition to this, the discretization error is another challenge that appears when reconstructing the signal, especially when moments are used in the implementation. This discretization error, however, increases with the moments’ order. For example, when the order of the moments increases, the discretization error increases accordingly. As such, the accuracy of the moments’ computation is reduced, resulting in an inaccurate representation of images [38,39,40].

Several studies have been performed on the discrete Krawtchouk polynomials and methods developed to efficiently compute their coefficients, for example see [25,31,41,42,43,44]. These research works utilize a three-term recurrence algorithm [25]. In addition, the hypergeometric series and gamma functions are widely applied in image processing [25]. However, the aforementioned research works use functions that require a long time to execute and process the signals. Furthermore, these functions become numerically unstable when the order of the moments increases. Instead, a three-terms recurrence algorithm can be applied to come up with the aforementioned time and accuracy issues. To this end, Yap et al. [41] presented a recurrence algorithm in the *n* direction to calculate the Krawtchouk polynomial coefficients (KPCs). Due to the propagation error, this recurrence algorithm becomes unstable—especially when the polynomial size increases. In general, such a propagation error increases through the computation of polynomial coefficients. This is attributed to the fact that pitfalls may happen even when small errors in floating numbers occur. As such, there is an essential need to reduce the number of recurrences, especially when the polynomial size is increased. Furthermore, such a reduction could also lead to a reduction in the propagation error, thereby leading to a more stable computation of polynomial coefficients, as desired. The work in [31] proposes a modified recurrence algorithm in the *n* direction (RAN) by partitioning the KP array into two partitions. Therefore, only 50% of the coefficients need to be computed. However, the partitioning of the KP array generates a larger polynomial size, which is undesirable. On a similar basis, the work in [42] proposes a recurrence algorithm in the *x* direction (RAX) by partitioning the KP plane into two partitions. Specifically, the *x* direction of the recurrence algorithm is used to compute the KPCs. The results show that the RAX algorithm outperforms the RAN algorithm. It is worth noting that the RAN and RAX algorithms use a symmetric property to compute the polynomial coefficients of the second portion. A novel bi-recursive relation algorithm in the n direction (BRRN) was proposed in [43]. In this method, the KP array is divided into four partitions. However, the KPC coefficients are computed for two partitions only, i.e., 50% of the coefficients are computed. Then, a symmetric property is used to compute the KPCs for the remaining partitions. The results indicate that the BRRN algorithm provides higher gain than the RAX algorithm for limited values of parameter *p*, i.e., the polynomial size. Abdulhussain et al. [44] developed an algorithm and presented new properties of orthogonal polynomials such that the KP plane is divided into four portions and only the KPCs for one portion are computed. For this, the size of the generated polynomials is increased, but it is still limited, especially for parameter *p* less than 0.25 and greater than 0.75. This is because the initial values or sets become zero as the polynomial size increases. Recently, the work in [25] proposed a recurrence relation algorithm that has the ability to compute KPCs with very large sizes. However, the proposed algorithm is limited to the parameter value of p=0.5.

The existing algorithms suffer from the following limitations: (1) no initial value is provided; (2) the propagation error is high; and (3) the implementation of these algorithms is limited to a specific value of the parameter *p*. They also suffer from numerical instabilities, especially when the polynomials orders and sizes become high. Therefore, an advanced and reliable recurrence algorithm for high-order polynomials and large sizes is required. Therefore, a new recurrence algorithm is presented in this paper, which handles the numerical instabilities issue of using high orders of polynomials and large sizes. The proposed algorithm is able to compute the KPCs for all values of the parameter *p*. In addition to this, this paper presents the development of a new mathematical model for computing the initial value of *p*. In particular, the initial value is accurately computed for all values of parameter *p*. Furthermore, a new relation to compute the values of the initial sets is derived. To this end, a diagonal recurrence relation is introduced. The proposed diagonal recurrence algorithm is derived from the existing *n* and *x* directions of the recurrence algorithm. The diagonal and the existing recurrence algorithm are exploited to compute the KP coefficients. The KP coefficients are then used for one partition after dividing the KP plane into four partitions. To compute the KP coefficients in other partitions, a symmetric property relation is utilized.

**Organization of the paper**: This paper is organized as follows. Section 2 presents the mathematical formulations of the orthogonal polynomials and moments. In Section 3, the methodology of the proposed recurrence algorithm is provided. This methodology involves providing a discussion about the initial value selection of parameter *p*. In addition, this section explains how the Krawtchouk polynomial’s coefficients can be computed. In order to characterize the performance of the proposed approaches, Section 4 provides the numerical results. Finally, conclusions are discussed in Section 5.

*Notation*: In this paper, the operator transpose is denoted by ${\left(\cdot \right)}^{T}$ and ab denotes the binomial coefficients.

## 2. Preliminaries

This section presents the Krawtchouk polynomials and their recurrence relation. To this end, the *n*-th order of the Krawtchouk polynomials based on the hypergeometric series is given as

(1) K^np(x)=2F1−n−x−N+1;1p.

The weighted function ω(x,p) and the norm function ρ(n,p) are used to generate the weighted and normalized KP coefficients as given in [31]. To this end, the weighted and normalized KP coefficients can be written as in (2) and (3), respectively:

(2) ω(x,p)=N−1xpx(1−p)N−x−1

(3) $$\begin{array}{cc} \rho (n,p) & ={\left(-1\right)}^{n}\;{\left(\frac{1-p}{p}\right)}^{n}\frac{n!}{{\left(-N+1\right)}_{n}} \end{array}$$

The Pochhammer symbol ${\left(\cdot \right)}_{c}$, which is known as an ascending or rising factorial function, can be written as [45]

(4) $${\left(a\right)}_{c}=\frac{\Gamma (a+c)}{\Gamma (a)}=a\left(a+1\right)\left(a+2\right)\cdots \left(a+c-1\right),$$

where Γ(.) denotes the Gamma function. To this end, using the weight and norm functions, the weighted and normalized Krawtchouk polynomials of the *n*-th order for a signal of size *N* are given as [31]

(5) $$\begin{array}{cc} K_{n}^{p}\left(x\right) & =\sqrt{\frac{\omega (n,x)}{\rho (n,x)}}\;{\hat{K}}_{n}^{p}\left(x\right), \end{array}$$

(6) Knpx=N−1nN−1xp1−pn+x2F1−n−x−N+1;1p,n,x=0,1,⋯,N−1;p∈(0,1),

where ${}_{2}F_{1}$ describes the hypergeometric series and can be written as

(7) 2F1−n−x−N+1;1p=∑k=0∞(−n)k(−x)k(−N+1)k,k!1pk.

## 3. Proposed Recurrence Algorithm

This section describes the methodology of the proposed recurrence algorithm.

### 3.1. Computing the Initial Value

The problem with traditional approaches for computing the initial value in the n=0 and x=0 directions is the numerical instability. For example, the traditional methods provide zero values of the initial $K_{0}^{p}\left(0\right)$—which is unstable. The initial value can be computed as

(8) $$K_{0}^{p}\left(0\right)=\sqrt{{\left(1-p\right)}^{N-1}}.$$

**Figure 1:**
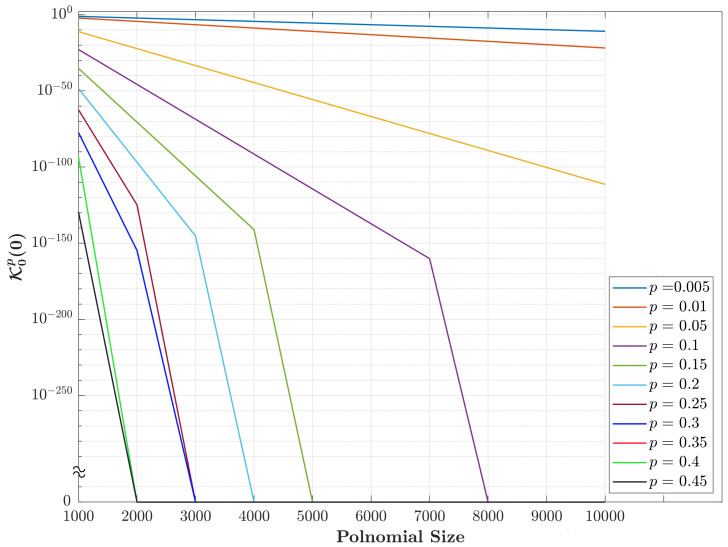
The computation of the initial values ($K_{0}^{p}\left(0\right)$) as a function of different polynomial sizes using Equation (8).

**Figure 2:**
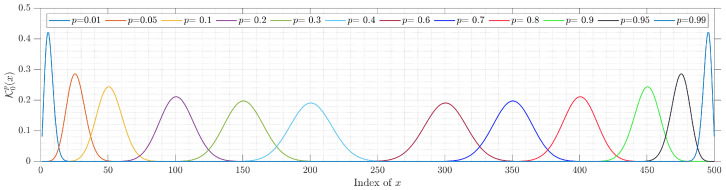
Plots of $K_{0}^{p}\left(x\right)$ for a wide range of parameter *p* and N=500.

The expression in (8) makes the initial value ($K_{0}^{p}\left(0\right)$) decrease to zero for different values of parameter *p*, especially for large polynomial sizes *N*. Figure 1 shows the values of $K_{0}^{p}\left(0\right)$ for different values of parameter *p* and size *N*. The results show that the value of $K_{0}^{p}\left(0\right)$ starts to fall to zero when *p* becomes larger than 0.1. Specifically, as the values of parameter *p* increase, the value of $K_{0}^{p}\left(0\right)$ falls to zero. For example, for p=0.15, the value of $K_{0}^{p}\left(0\right)$ becomes zero for N>5000 while for p=0.4, the value of $K_{0}^{p}\left(0\right)$ becomes zero earlier for N>2000. This makes it impossible to compute the rest of the KP coefficients’ values. Therefore, there is an essential need to find an efficient method for computing the initial value of *p*, which prevents the initial value ($K_{0}^{p}\left(0\right)$) from dropping to zero. To this end, this paper identifies the suitable non-zero values in the KP plane that need to be used as an initial value. As such, there is an essential need to plot the values of coefficient $K_{0}^{p}\left(x\right)$ for different values of parameter *p*. Figure 2 shows the plots of the values of coefficient $K_{0}^{p}\left(x\right)$ for different values of parameter *p*. Clearly, the results in Figure 2 show that the values of $K_{0}^{p}\left(x\right)$ start with a small number and gradually reach the peak. Then, the values drop to a very small number. In addition, we observe that using non-small values as an initial set of parameter *p* seems useful to compute other values of the KP coefficients.

Figure 2 demonstrates that the peak values can be located at x=Np. In this paper, the value of x=Np is denoted by $x_{0}$. To this end, a general formula for computing $K_{0}^{p}\left(x_{0}\right)$ can be written as

(9) K0px0=K0p(Np)×ω(Np;p)ρ(0;p),K0px0=1×N−1NppNp1−p−Np+N−11,K0px0=N−1NppNp1−p−Np+N−1.

**Figure 3:**
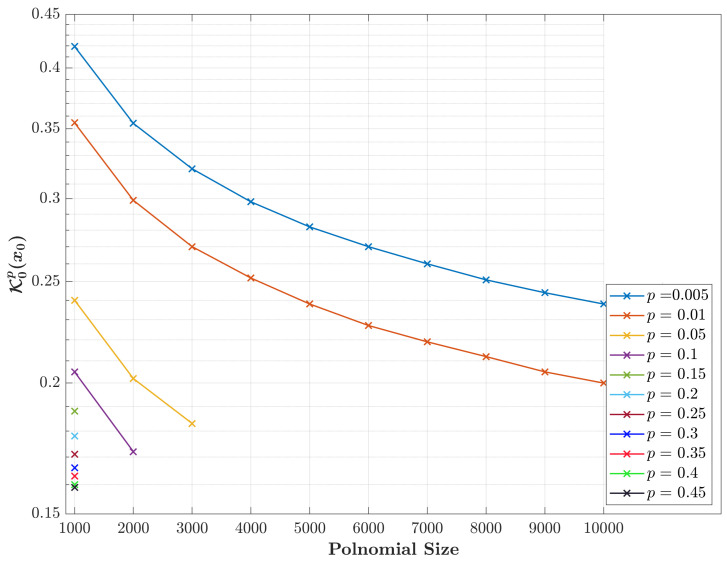
The computation of the initial values ($K_{0}^{p}\left(x_{0}\right)$) as a function of different polynomial sizes using Equation (9).

Computing $K_{0}^{p}\left(x_{0}\right)$ using expression (9) may also lead to unstable numerical values of coefficients with errors, especially for high-order polynomials. This is because the binomial coefficients function tends to be very large and close to infinity. To demonstrate this behavior, Figure 3 is provided.

Figure 3 shows that the initial values ($K_{0}^{p}\left(x_{0}\right)$) are still inaccurate where these values record either NaN or Inf values. This is due to the nature of the polynomial coefficients that are obtained by the expression in (9). Thus, the initial values ($K_{0}^{p}\left(x_{0}\right)$) seem difficult to be computed for large polynomial sizes (*N*). To overcome this issue, this paper proposes an efficient and suitable approach that makes the value of $K_{0}^{p}\left(x_{0}\right)$ commutable. It is worth noting that the values obtained from the polynomial coefficients’ formula, especially the Gamma function, should be reduced when the coefficients become large since their argument value is increased. This can be achieved using $\arg=\exp\left(\ln\left(arg\right)\right)=e^{\ln\left(\arg\right)}$ and the initial values can be computed as

(10) $$\begin{array}{c} K_{0}^{p}\left(x_{0}\right)=e^{0.5\times z}, \end{array}$$

where *z* is given as

$$\begin{array}{cc} z & =\ln\Gamma \left(N\right)-\log\Gamma \left(x_{0}+1\right)-\log\Gamma \left(N-x_{0}\right)-x_{0}\ln\left(\frac{1-p}{p}\right)+\left(N-1\right)\ln\left(1-p\right). \end{array}$$

A proof of expression (10) is presented in Appendix A.

**Figure 4:**
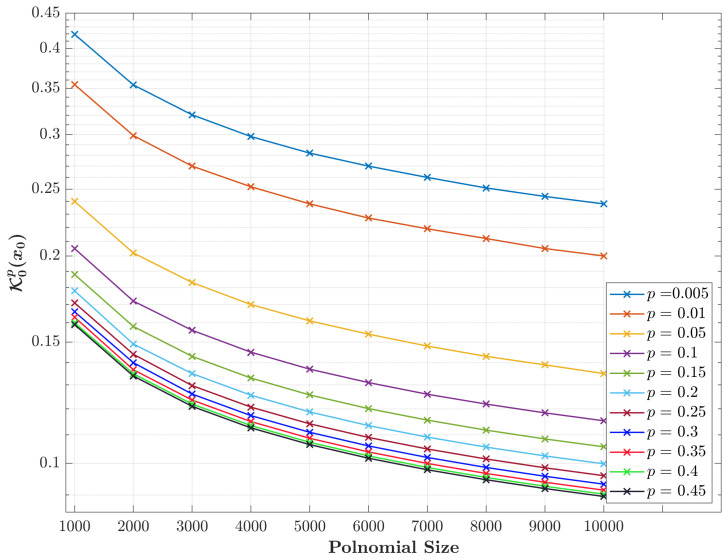
The computation of the initial sets’ values ($K_{0}^{p}\left(x_{0}\right)$) as a function of different polynomial sizes using Equation (10).

Figure 4 shows a plot of the proposed initial values of ($K_{0}^{p}\left(x_{0}\right)$) in the developed expression in (10) for various values of parameter *p* as a function of polynomials size *N*. The results show that the proposed initial values are more computable for wide ranges of parameter *p* and large polynomial sizes *N*, as desired. Hence, this signifies the feasibility of the proposed formula for practical implementations compared with state-of-the-art equations.

### 3.2. The Fundamental Computation of the Initial Values

**Figure 5:**
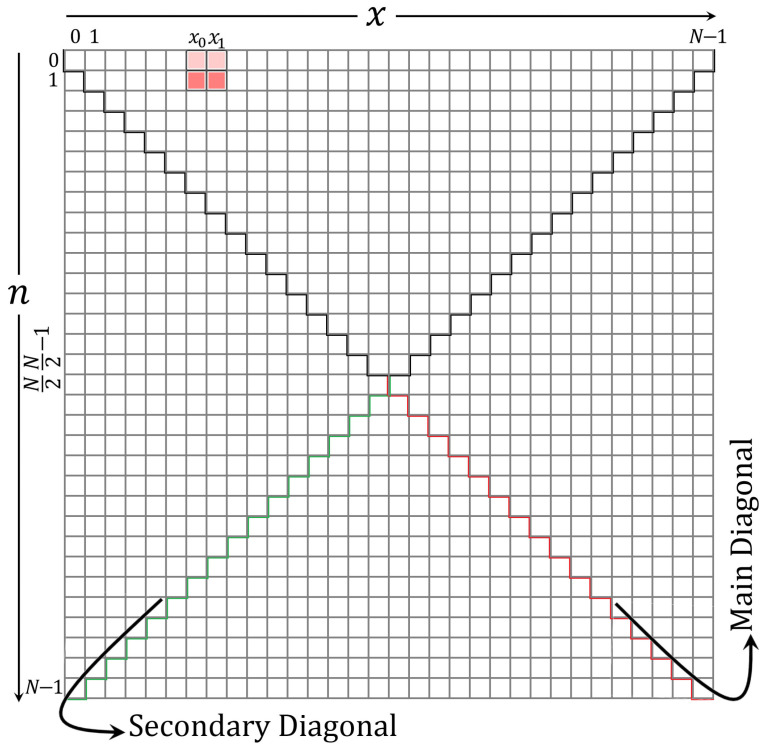
The fundamental computation of initial values according to the *x* and *n* directions in the KP plane.

Typically, for any orthogonal polynomial, the computation of coefficients requires the evaluation of a significant number of fundamental initials. Thus, based on the first initial value $K_{0}^{p}\left(x_{0}\right)$, computed using the proposed formula, the KP coefficients are obtained $K_{0}^{p}\left(x_{1}\right)$, $K_{1}^{p}\left(x_{0}\right)$, and $K_{1}^{p}\left(x_{1}\right)$ (see Figure 5). Therefore, this section shows how the aforementioned coefficients values are computed.

First, $K_{0}^{p}\left(x_{1}\right)$ is computed using the proposed derived formula, which provides the two terms relation between the $K_{0}^{p}\left(x_{0}\right)$ and $K_{0}^{p}\left(x_{1}\right)$. To this end, this relation/ratio between the coefficients can be formulated as

(11) K0p(x1)K0p(x0)=N−1Np+1pNp+1(1−p)−Np+N−2N−1NppNp(1−p)−Np+N−1,=(N−1)!(Np+1)!(N−Np−2)!(N−1)!(Np)!(N−Np−1)!·1−pp,=N−Np−1Np+1·p1−p,

where $x_{1}=x_{0}+1=Np+1$. Thus, the expression in (11) can be further simplified to:

(12) $$K_{0}^{p}\left(x_{1}\right)=\sqrt{\frac{N-Np-1}{Np+1}\cdot \frac{p}{1-p}}\;\;\;\;K_{0}^{p}\left(x_{0}\right).$$

Then, $K_{1}^{p}\left(x_{0}\right)$ and $K_{1}^{p}\left(x_{1}\right)$ can be computed using a two-term recurrence relation with $K_{0}^{p}\left(x_{0}\right)$ and $K_{0}^{p}\left(x_{1}\right)$, respectively. To derive the recurrence relation of the proposed approach, the following formulas are used:

(13) $$\begin{array}{cc} K_{0}^{p}\left(x\right) & =\sqrt{\omega_{K}^{p}\left(x,N\right)}, \end{array}$$

(14) $$\begin{array}{cc} K_{1}^{p}\left(x\right) & =\sqrt{\omega_{K}^{p}\left(x,N\right)},\frac{p(N-1)-x}{\sqrt{\left(N-1)p(1-p\right)}}. \end{array}$$

From (13) and (), $K_{1}^{p}\left(x\right)$ can be simplified to:

(15) $$K_{1}^{p}\left(x\right)=\frac{p(N-1)-x}{\sqrt{\left(N-1)p(1-p\right)}}\;\;\;K_{0}^{p}\left(x\right).$$

Using the expression in (15), $K_{1}^{p}\left(x_{0}\right)$ and $K_{1}^{p}\left(x_{1}\right)$ can be further simplified to

(16) $$\begin{array}{cc} K_{1}^{p}\left(x_{0}\right) & =\frac{p(N-1)-Np}{\sqrt{\left(N-1)p(1-p\right)}}\;\;\;K_{0}^{p}\left(x_{0}\right)\\ \; & =\frac{p}{\sqrt{\left(N-1)p(1-p\right)}}\;\;\;K_{0}^{p}\left(x_{0}\right) \end{array}$$

(17) $$\begin{array}{cc} K_{1}^{p}\left(x_{1}\right) & =\frac{p(N-1)-(Np+1)}{\sqrt{\left(N-1)p(1-p\right)}}\;\;\;K_{0}^{p}\left(x_{1}\right)\\ & =\frac{p+1}{\sqrt{\left(N-1)p(1-p\right)}}\;\;\;K_{0}^{p}\left(x_{1}\right). \end{array}$$

### 3.3. The Computation of the Initial Sets

**Figure 6:**
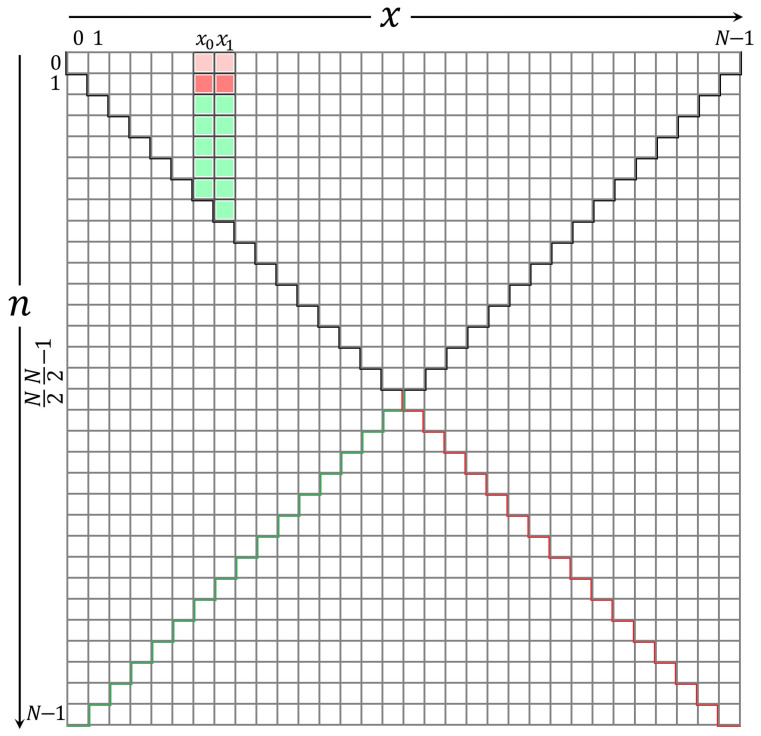
A diagram shows the location of initial sets in the KP plane.

In this section, the computation of the initial sets is discussed. These initial sets are shown in Figure 6. Figure 6 shows parts of the KP coefficients, which are covered in this section. The initial sets are defined in the ranges of $x=x_{0},x_{1}$ and n=2,3,⋯,x.

To compute the values of the initial set, the recurrence in the *n* direction is used. To this end, the formulation of recurrence is given as

(18) $$\begin{array}{cc} K_{n}^{p}\left(x\right) & =\alpha_{1_{n,x}}\;K_{n-1}^{p}\left(x\right)-\alpha_{2_{n,x}}\;K_{n-2}^{p}\left(x\right),\\ \alpha_{1_{n,x}} & =\frac{(N-2n+1)p+n-x-1}{\sqrt{p(1-p)n(N-n)}},\\ \alpha_{2_{n,x}} & =\sqrt{\frac{\left(n-1)(N-n+1\right)}{n(N-n)}},\\ x=\; & x_{0},x_{1}\;\mathrm{and}\;n=2,3,\cdots ,x. \end{array}$$

After computing the initial sets, the values in the ranges $x=x_{0},x_{1}$ and n=0,1,⋯,x are used as the initials to compute the rest of the KP coefficients values.

### 3.4. Computation of the Coefficients Values for KP

**Figure 7:**
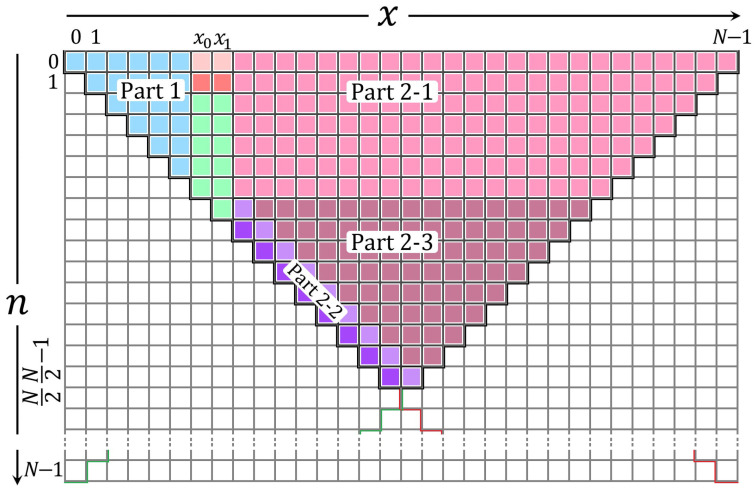
A diagram shows the parts’ locations in the KP coefficients’ plane in the *x* and *n* directions based on the proposed algorithm.

In this section, the rest of the coefficient values are computed. These coefficients are shown in Figure 7. As depicted in Figure 7, there are two main parts, which are located at the left (Part 1) and the right (Part 2) sides of the initial sets. In addition, the coefficients are located at the right side of the initial sets and can be divided into three sub-parts. These parts are Part 2-1, Part 2-2, and Part 2-3. The detailed description of the computation of each part is presented in the following subsections.

#### 3.4.1. Computation of the Coefficients Located at Part 1

In this section, the values of KP coefficients in Part 1, shown in Figure 7, are computed using a backward *x* recurrence relation. The backward recurrence relation is obtained from the traditional recurrence relation in the *x* direction as

(19) $$\begin{array}{cc} K_{n}^{p}\left(x-1\right) & =\beta_{1_{n,x}}\;K_{n}^{p}\left(x\right)-\beta_{2_{n,x}}\;K_{n}^{p}\left(x+1\right),\\ \beta_{1_{n,x}} & =-\frac{(N-2x-1)p-n+x}{\sqrt{p(1-p)x(N-x)}},\\ \beta_{2_{n,x}} & =\sqrt{\frac{\left(N-x-1)(x+1\right)}{x(N-x)}},\\ n=\; & 0,1,\cdots ,x_{0}\;\mathrm{and}\;x=x_{0},x_{0}-1,\cdots ,n. \end{array}$$

The values of KP coefficients become unstable as the index of *x* goes towards *n*. This is because the values of the coefficients tend to be less than $10^{-7}$. To overcome this issue, the condition of a threshold value is used to stop the recurrence for each value of index *n*. The proposed condition is given by

(20) $$|K_{n}\left(x\right)|<10^{-5}\;\mathrm{and}\;\left|K_{n}\left(x+1\right)\right|<10^{-7}.$$

#### 3.4.2. Computation of the Coefficients Located at Part 2-1

In this section, the values of KP coefficients in Part 2-1, given in Figure 7, were computed using a forward *x* recurrence relation as given in (21):

(21) $$\begin{array}{cc} K_{n}^{p}\left(x+1\right) & =\gamma_{1_{n,x}}\;K_{n}^{p}\left(x\right)-\gamma_{2_{n,x}}\;K_{n}^{p}\left(x-1\right)\\ \gamma_{1_{n,x}} & =\frac{(N-2x-1)p-n+x}{\sqrt{p(1-p)(x+1)(N-x-1)}}\\ \gamma_{2_{n,x}} & =-\sqrt{\frac{(N-x)x}{\left(x+1)(N-x-1\right)}}\\ n=\; & 0,1,\cdots ,x_{0}\;\mathrm{and}\;x=x_{0},x_{0}+1,\cdots ,N-n-1 \end{array}$$

The aforementioned recurrence relation, which is used to compute the values in Part 2-1, is subject to the following condition:

(22) $$|K_{n}\left(x\right)|<10^{-5}\;\mathrm{and}\;\left|K_{n}\left(x+1\right)\right|<10^{-7}.$$

#### 3.4.3. Computation of the Coefficients Located at Part 2-2

This section presents two new recurrence relations’ approaches to compute the KP coefficient values diagonally. This diagonal calculation is given in Part 2-2 Figure 7. The values in the diagonal of Figure 7 are then used to compute the coefficients’ values in Part 2-3 in Figure 7. This is because the recurrence relation in the *n* direction cannot be used to compute the coefficients’ values. Consequently, some values in Part 2 become zero, which results from the condition used to prevent the occurrence of unstable values.

**Figure 8:**
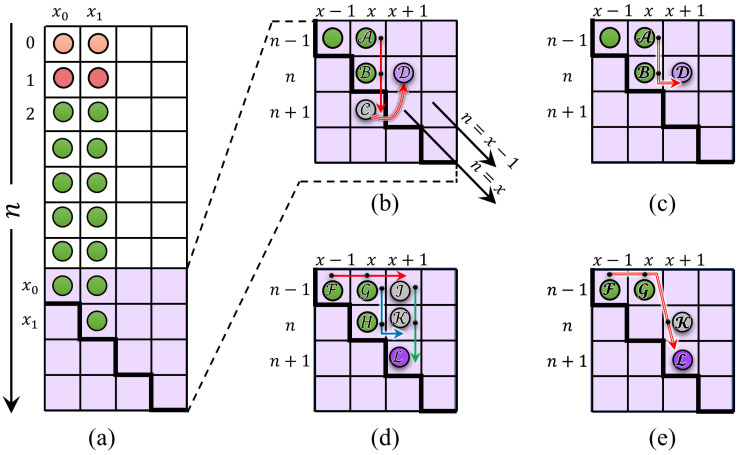
A diagram shows the coefficients’ locations that are used to compute the values in Part 2-2.

This paper derives the recurrence relations provided in Figure 8. From Figure 8a, it can be seen that the elements computed for $x_{0}$ and $x_{1}$ can be used to compute the coefficients along the main diagonal n=x and n=x−1. Furthermore, to compute the coefficients’ values of KP $K_{n}^{p}\left(x+1\right)$, the coefficient value $K_{n+1}^{p}\left(x\right)$ is computed using the *n* direction recurrence algorithm. The similarity across the main diagonal (n=x) is exploited for simplicity where $K_{n}^{p}\left(x+1\right)=K_{n+1}^{p}\left(x\right)$. To this end, the KP coefficients along n=x−1 are computed as

(23) $$\begin{array}{cc} K_{n}^{p}\left(x+1\right) & =\delta_{1_{n,x}},\;K_{n}^{p}\left(x\right)-\delta_{2_{n,x}},\;K_{n-1}^{p}\left(x\right),\\ \delta_{1_{n,x}} & =\frac{(N-2x-1)p-n+x}{\sqrt{p(1-p)x(N-x)}},\\ \delta_{2_{n,x}} & =-\sqrt{\frac{(N-x)x}{\left(x+1)(N-x-1\right)}},\\ x=\; & x_{0}+1,x_{0}+2,\frac{N}{2}-1\;\mathrm{and}\;n=x. \end{array}$$

To compute the values at the main diagonal where n=x, a new recurrence relation approach is developed. This is achieved by combining both *n* and *x* directions recurrences. Suppose that the values at (n,x+1),and(n−1,x+1) are known (see circulated values I and K in Figure 8d). Then, the value at n+1,x+1 (see circulated values L in Figure 8d) can be computed using the *n* direction recurrence relation as

(24) $$\begin{array}{c} K_{n+1}^{p}\left(x+1\right)=\alpha_{1_{n+1,x+1}},\;K_{n}^{p}\left(x+1\right)-\alpha_{2_{n+1,x+1}},\;K_{n-1}^{p}\left(x+1\right). \end{array}$$

The value at (n−1,x+1) can be computed using the *x* direction recurrence relation as

(25) $$\begin{array}{c} K_{n-1}^{p}\left(x+1\right)=\gamma_{1_{n-1,x}},\;K_{n-1}^{p}\left(x\right)-\gamma_{2_{n-1,x}},\;K_{n-1}^{p}\left(x-1\right). \end{array}$$

Substituting Equation (25) in (24) yields the following general expression of the recurrence relation:

(26) $$\begin{array}{cc} K_{n+1}^{p}\left(x+1\right) & =\alpha_{1_{n+1,x+1}}\;K_{n}^{p}\left(x+1\right)-\alpha_{2_{n+1,x+1}}\left(\gamma_{1_{n-1,x}}\;K_{n-1}^{p}\left(x\right)-\gamma_{2_{n-1,x}}\;K_{n-1}^{p}\left(x-1\right)\right)\\ \; & =\alpha_{1_{n+1,x+1}}\;K_{n}^{p}\left(x+1\right)-\alpha_{2_{n+1,x+1}}\gamma_{1_{n-1,x}}\;K_{n-1}^{p}\left(x\right)+\alpha_{2_{n+1,x+1}}\gamma_{2_{n-1,x}}\;K_{n-1}^{p}\left(x-1\right)\\ \; & =\eta_{1_{n,x}},K_{n}^{p}\left(x+1\right)-\eta_{2_{n,x}}\;K_{n-1}^{p}\left(x\right)+\eta_{3_{n,x}}\;K_{n-1}^{p}\left(x-1\right)\\ \eta_{1_{n,x}} & =\alpha_{1_{n+1,x+1}}=\frac{(N-2n-1)p+n-x-1}{\sqrt{p(1-p)(n+1)(N-n-1)}}\\ \eta_{2_{n,x}} & =\alpha_{2_{n+1,x+1}}\gamma_{1_{n-1,x}}=\sqrt{\frac{n\left(N-n\right){\left(\left(N-2x-1\right)p+x-n+1\right)}^{2}}{p(1-p)(n+1)(N-n-1)(x+1)(N-x-1)}}\\ \eta_{3_{n,x}} & =\alpha_{2_{n+1,x+1}}\gamma_{2_{n-1,x}}=\sqrt{\frac{n(N-n)x(N-x)}{\left(n+1)(N-n-1)(x+1)(N-x-1\right)}}\\ x & =x_{1},x_{1}+1,\cdots ,N/2-1;\;\mathrm{and}\;n=x \end{array}$$

**Figure 9:**
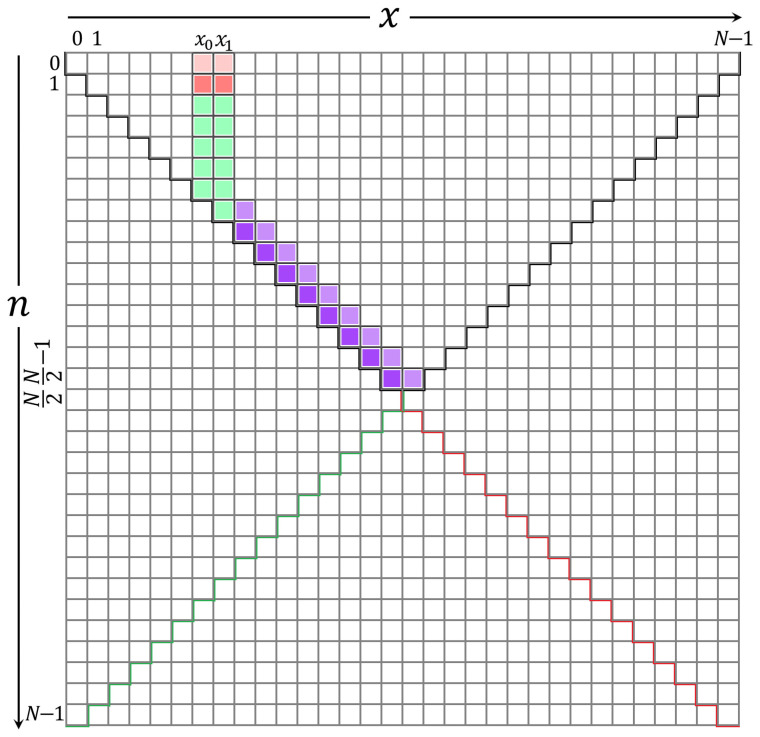
A diagram shows a location of the coefficients in Part 2-2.

This recurrence relation is termed as the four-term recurrence relation in the n—x direction. This new development approach is used to compute the KP coefficients in the range $x=x_{1}+1,x_{1}+2,\cdots ,N/2+1$ and n=x−1, and $x=x_{1}+1,x_{1}+2,\cdots ,N/2$ and n=x as shown in Figure 9:

#### 3.4.4. Computation of the Coefficients Located at Part 2-3

This section presents the computation of the KP coefficients located at Part 2-3 in Figure 7. These values are computed using (21) in the ranges $n=x_{1},x_{1}+1,N/2-2$ and n+2≤x≤N−n+1. However, the following condition should be met:

(27) $$|K_{n}\left(x\right)|<10^{-5}\;\mathrm{and}\;\left|K_{n}\left(x+1\right)\right|<10^{-7}.$$

### 3.5. Computation of the Rest of the KP Coefficients

This subsection provides the computation of the rest of the KP coefficients. To this end, the rest of the coefficients can be computed using a similarity relation of the KP. The coefficients in the ranges x=0,1,⋯,N/2−1 and n=x+1,x+2,⋯,N−x−1 are given as

(28) $$K_{n}^{p}\left(x\right)=K_{x}^{p}\left(n\right).$$

The coefficients in the ranges x=0,1,⋯,N−1 and n=N−x,N−x+1,⋯,N−1 are computed using the following expression:

(29) $$K_{n}^{p}\left(x\right)={\left(-1\right)}^{N-n-x-1}K_{N-n}^{p}\left(N-x\right).$$

In addition, to calculate the KP coefficients for p>0.5, firstly the value of *p* is set to 1−p. Then, the KP coefficients are computed using the proposed methodology. Finally, the following formula is applied for all coefficients [44]:

(30) $$K_{n}^{p}\left(x\right)={\left(-1\right)}^{n}K_{n}^{p}\left(N-x-1\right).$$

### 3.6. Summary of the Proposed Algorithm

**Figure 10:**
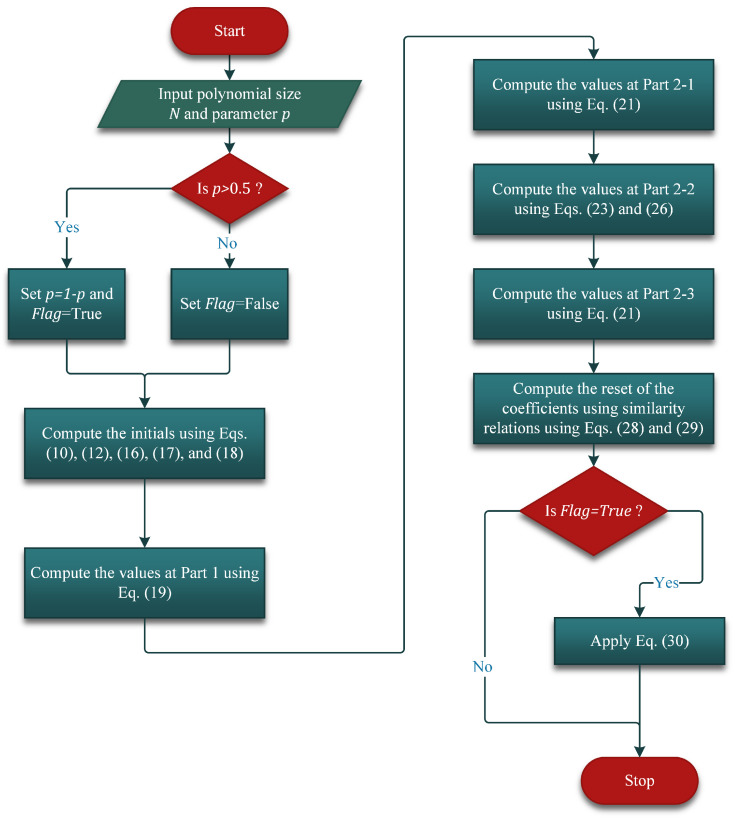
Flowchart of the proposed algorithm.

In this subsection, a summary of the proposed algorithm is presented. To this end, a flow chart of the proposed recurrence is shown in Figure 10. In addition to this, a pseudo-code is presented (see Algorithm 1) for more clarification. In addition, 3D plots of the KP coefficients are given in this subsection.
**Algorithm 1** Computation of Krawtchouk polynomials using the proposed algorithm.     **Input: N,p**                   *N* represents the size of the Krawtchouk polynomial,                   *p* represents the parameter of the Krawtchouk polynomials.     **Output: $K_{n}^{p}\left(x\right)$**1: Flag=False
2: **if**
 p>0.5 
**then**3:     Flag=True;       p←p−14: **end if**5: $x_{0}\leftarrow N\;p$,       $x_{1}\leftarrow x_{0}+1$6: Compute $K_{0}^{p}\left(x_{0}\right)$ using (10)7: Compute $K_{0}^{p}\left(x_{1}\right)$ using (12)8: Compute $K_{1}^{p}\left(x_{0}\right)$ and $K_{1}^{p}\left(x_{1}\right)$ using (16) and (17)9:▹ Compute initial set10: **for**
$x=x_{0}:x_{1}$
**do**11:     **for** n=2:x **do**12:         Compute $K_{n}^{p}\left(x\right)$ using (18)13:     **end for**14: **end for**15:▹ Compute coefficient values in Part 116: **for**
$n=0:x_{0}$
**do**17:     **for** $x=x_{0}:-1:n$**do**▹ inner loop18:         Compute $K_{n}^{p}\left(x\right)$ using (19)19:         **if** $|K_{n}\left(x\right)|<10^{-5}\;\mathrm{and}\;\left|K_{n}\left(x+1\right)\right|<10^{-7}$ **then**20:            Exit inner loop21:         **end if**22:     **end for**23: **end for**24:▹ Compute coefficient values in Part 2-125: **for**
$n=0:x_{0}$
**do**26:     **for** $x=x_{0}:N-n-1$**do**▹ inner loop27:         Compute $K_{n}^{p}\left(x\right)$ using (21)28:         **if** $|K_{n}\left(x+1\right)|<10^{-7}\;\mathrm{and}\;\left|K_{n}\left(x\right)\right|<10^{-5}$ **then**29:            Exit inner loop30:         **end if**31:     **end for**32: **end for**33:▹ Compute coefficient values in Part 2-234: **for**
$x=x_{0}:N/2-1$
**do**35:     n←x36:     Compute $K_{n}^{p}\left(x\right)$ using (23)37: **end for**38: **for**
$x=x_{1}:N/2-1$
**do**39:     n←x40:     Compute $K_{n}^{p}\left(x\right)$ using (26)41: **end for**42:▹ Compute coefficient values in Part 2-343: **for**
$n=x_{1}:N/2-2$
**do**44:     **for** x=n+2:N−n−1**do**▹ inner loop45:         Compute $K_{n}^{p}\left(x\right)$ using (21)46:         **if** $|K_{n}\left(x\right)|<10^{-5}\;\mathrm{and}\;\left|K_{n}\left(x+1\right)\right|<10^{-7}$ **then**47:            Exit inner loop48:         **end if**49:     **end for**50: **end for**51: Compute the rest of the coefficients using the similarity relations (28) and (29)52: **if**
Flag=True **then**53:     Apply (30)54: **end if**


**Figure 11:**
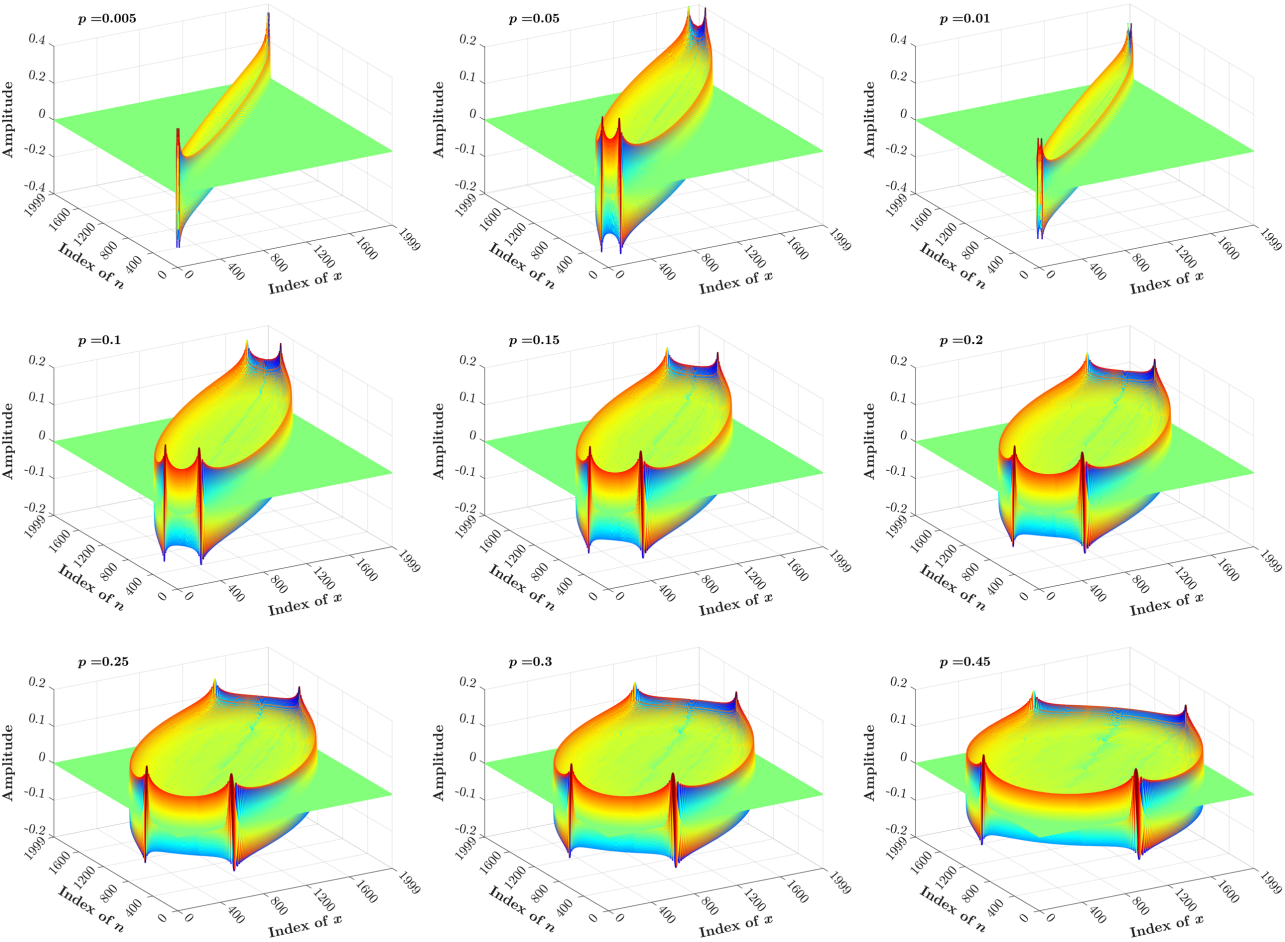
3D plot of the KP coefficients computed for N=2000 and p<0.5.

**Figure 12:**
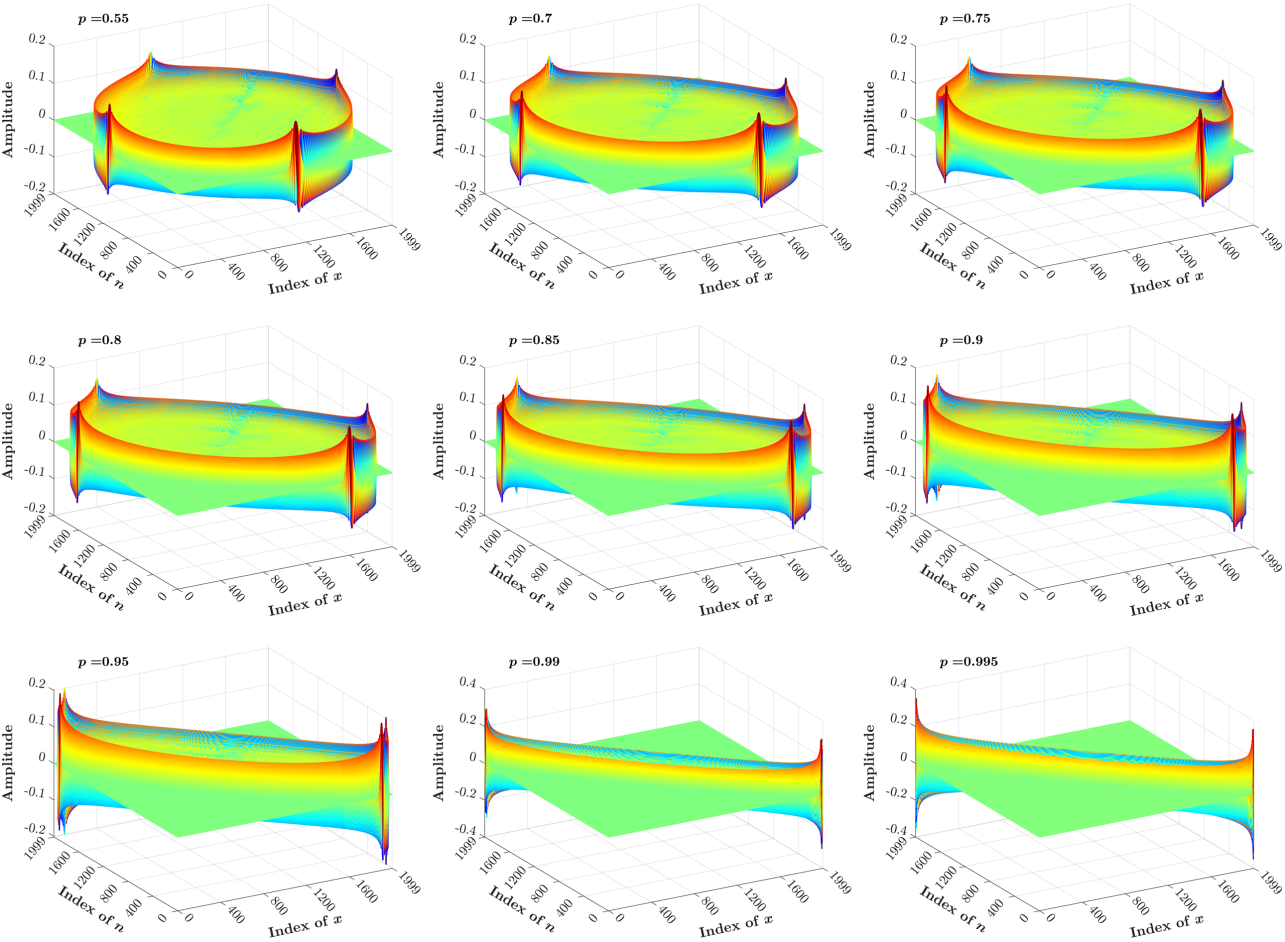
3D plot of the KP coefficients computed for N=2000 and p>0.5.

Figure 11 and Figure 12 show a 3D plot of the KP coefficients, which are generated using the proposed recurrence algorithm with N=2000 and different values of the *p* parameter ranging between <0.5 and >0.5, respectively.

## 4. Numerical Results and Analyses

This section presents the results obtained using the proposed recurrence algorithm. In addition, a comprehensive comparison is conducted with the existing recurrence algorithms. The comparison is carried out in terms of the energy compaction, reconstruction error, and computation cost.

### 4.1. Energy Compaction Analysis

The order of moments *n* impacts the process of signal reconstruction, energy compaction, and information retrieval. The order of the KP moments is given by n=0,1,2,⋯,N−1. The energy compaction is utilized to check the impact of using KP to transform a large fraction of the signal energy into relatively few coefficients of moments. To find the impact of using the KP parameter (*p*) on the energy compaction property, the procedure given by [46] is employed. The stationary Markov sequence with length *N* and zero mean is analyzed. A matrix L with covariance coefficients (ρ) is defined as [27]:

(31) $$L=\left[\begin{array}{cccc} 1 & \rho & \cdots & \rho^{N-1}\\ \rho & 1 & \cdots & \vdots \\ \vdots & \ddots & \ddots & \vdots \\ \rho^{N-1} & \cdots & \rho & 1 \end{array}\right]$$

The matrix L is then transformed to the Krawtchouk domain. As such, the coefficients in the main diagonal of the transformed matrix (S) are computed. The matrix S represents the variance $\sigma_{l}^{2}$, which can be computed as

(32) $$S=R\;L\;R^{T},$$

where *R* denotes the KP matrix and ${\left(\cdot \right)}^{T}$ refers to the matrix transpose operation. In addition, the normalized restriction error ($J_{m}$) can be computed using:

(33) $$\begin{array}{c} J_{m}=\frac{\sum_{q=m}^{N-1}\sigma_{q}^{2}}{\sum_{q=0}^{N-1}\sigma_{q}^{2}}, \end{array}$$

(34) $$\begin{array}{c} m=0,1,\cdots ,N-1, \end{array}$$

where $\sigma_{q}^{2}$ represents the order of $\sigma_{l}^{2}$ sorted in descending order. In the experiment, the normalized restriction error is performed by considering different covariance coefficients (ρ) and different values of the parameters MNP.

**Figure 13:**
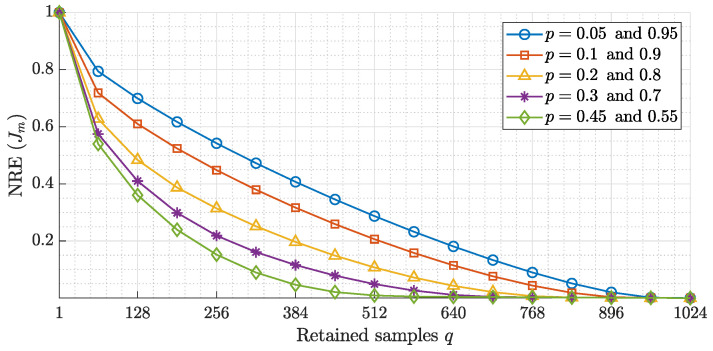
Energy compaction for different values of the parameter *p*.

Figure 13 shows the normalized restriction error for different values of parameters (*p*) with the covariance coefficient ρ=0.93. It can be observed from Figure 13 that when the value of *p* is equal to 1−p, the normalized restriction error becomes equal. For example, the normalized restriction error for p=0.05 is equal to p=1−0.05=0.95. In addition, the energy compaction is influenced by the KP parameter *p*. For instance, as the parameter *p* increases from 0.05 to 0.45, the performance of the KP in terms of energy compaction is changed. Furthermore, the energy compaction at parameter p=0.45 shows better performance for parameter p=0.05 because the normalized restriction error ($J_{m}$) reaches zero values. However, small values of parameter *p* shows better performance in terms of feature extraction, as proven in [44]. Thus, it can be concluded that KP provides further performance improvement as the parameter *p* reaches 0.5. Furthermore, a more accurate result can be achieved when the parameter *p* is deviates from 0.5 [44].

### 4.2. Analysis of Reconstruction Error

**Figure 14:**
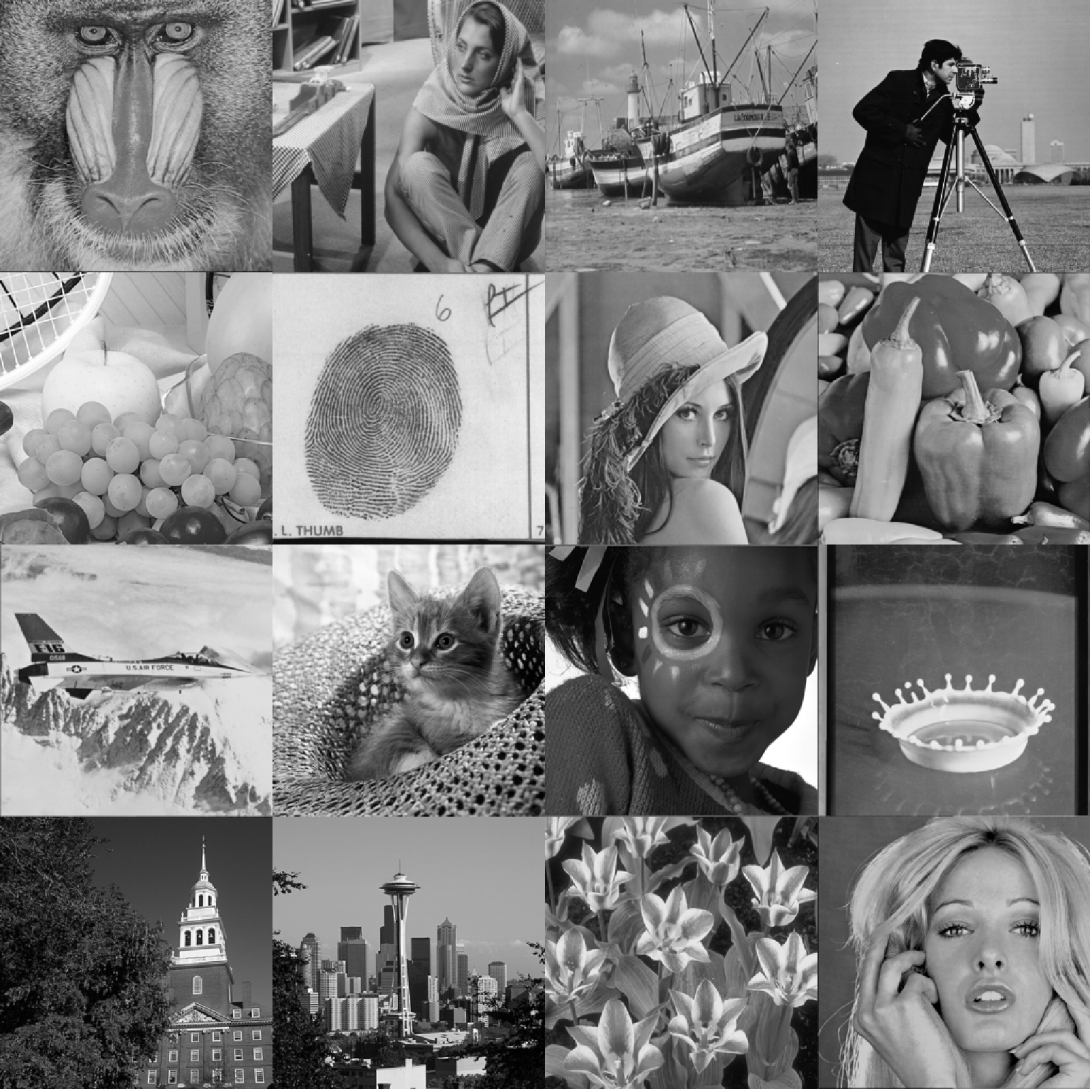
Evaluation of an image used for REA.

In this section, the proposed recurrence algorithm is evaluated by carrying out reconstruction error analysis (REA). This REA was conducted for the proposed and the existing works. The REA was performed using an image formed from 16 well-known images as shown in Figure 14. In addition, the comparison was performed on an image with a large size, i.e., (4096×4096). Different values of parameter *p* were considered in the analysis. These values are p=0.1,0.2,0.3,and0.4.

First, the WNKP (*R*) is generated using the proposed and existing algorithms. Then, the KMs (ψ) of the image are computed. Then, the image is reconstructed from the computed moments using a limited number of moments. Finally, the normalized mean square error (NMSE) is calculated between the input image and the reconstructed version of the image. Hence, the NMSE is given as [44]

(35) $$\mathrm{NMSE}\left(I,I_{Rec}\right)=\frac{\sum_{x,y}{\left(I\left(x,y\right)-I_{Rec}\left(x,y\right)\right)}^{2}}{\sum_{x,y}{\left(I(x,y)\right)}^{2}},$$

where parameters *I* and $I_{Rec}$ denote the original image and the reconstructed image, respectively.

**Figure 15:**
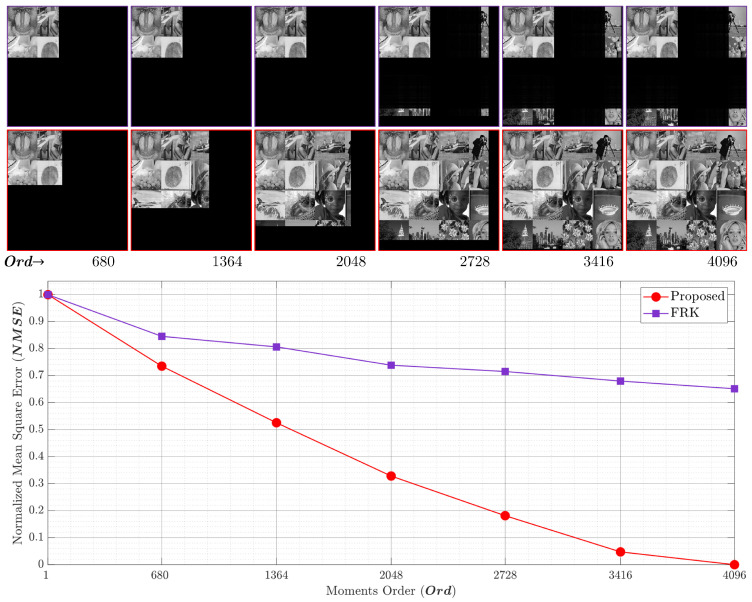
The NMSE performance comparing the proposed algorithm and the RAK algorithm [44] with p=0.1.

**Figure 16:**
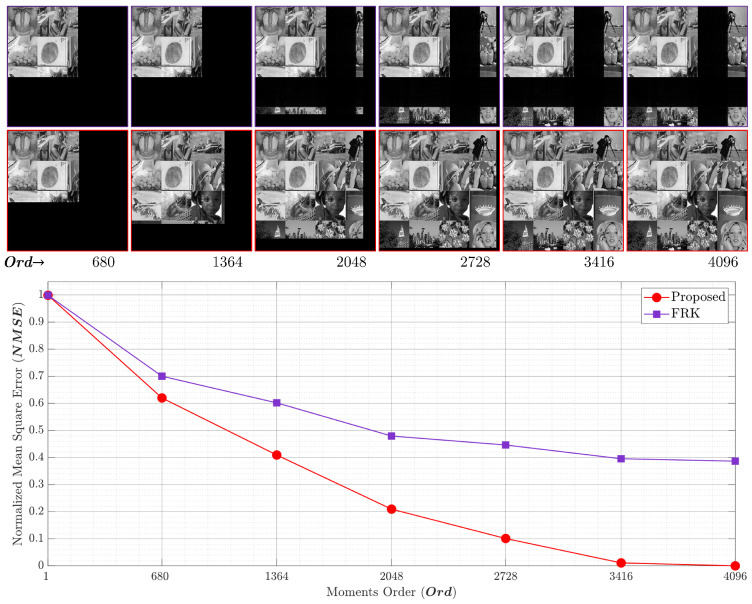
The NMSE performance comparing the proposed algorithm and the RAK algorithm [44] with p=0.2.

**Figure 17:**
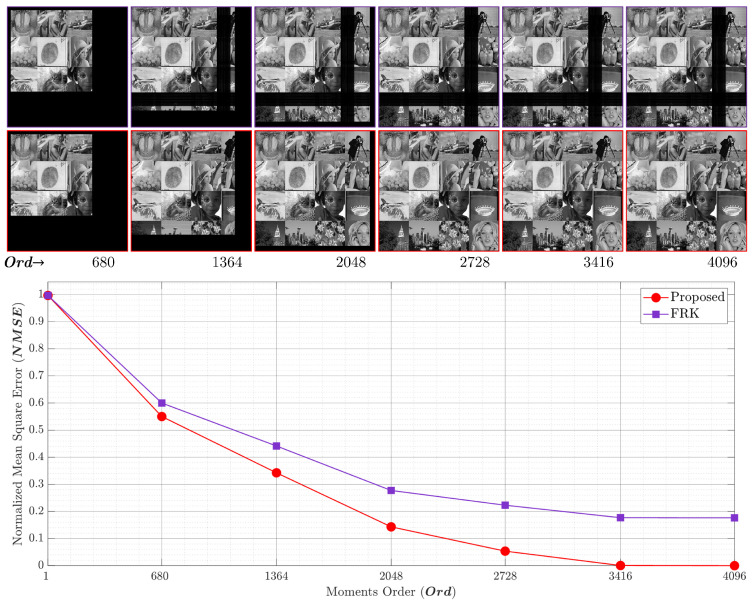
The NMSE performance comparing the proposed algorithm and the RAK algorithm [44] with p=0.3.

The NMSE and the reconstructed image for p=0.1andp=0.2 are shown in Figure 15 and Figure 16, respectively. The first row depicts the reconstructed images by utilizing the FRK [44], while the second row represents the reconstructed images using the proposed algorithm. The FRK algorithm is unable to reconstruct the image of the low-order moments. In addition, it is unable to reconstruct the image with high orders. However, the proposed algorithm is capable of fully reconstructing the image of different order values. In addition, the NMSE is minimized in the proposed algorithm using a moment order of 680, which is ∼16%. Figure 15 shows that the proposed algorithm achieves an NMSE of 0.72 while the FRK algorithm records a value of 0.84. This implies that the proposed algorithm outperforms the FRK algorithm. Moreover, the NMSE reaches zero when the proposed algorithm is used, while it records 0.64 when the FRK is used. The limitation with the FRK algorithm is due to the initial set that was computed, which makes the KPCs values tend towards zero, and thus, the NMSE is increased. Figure 17 provides a plot of experiments using p=0.3. The results show that the proposed algorithm provides better NMSE than the FRK starting from a moment order of 680. In addition, the performance improvement increases when the moment order increases until it reaches the full order of (4096). At the full order, the NMSE using the proposed algorithm reaches 0, while it reaches 0.18 using the FRK algorithm.

**Figure 18:**
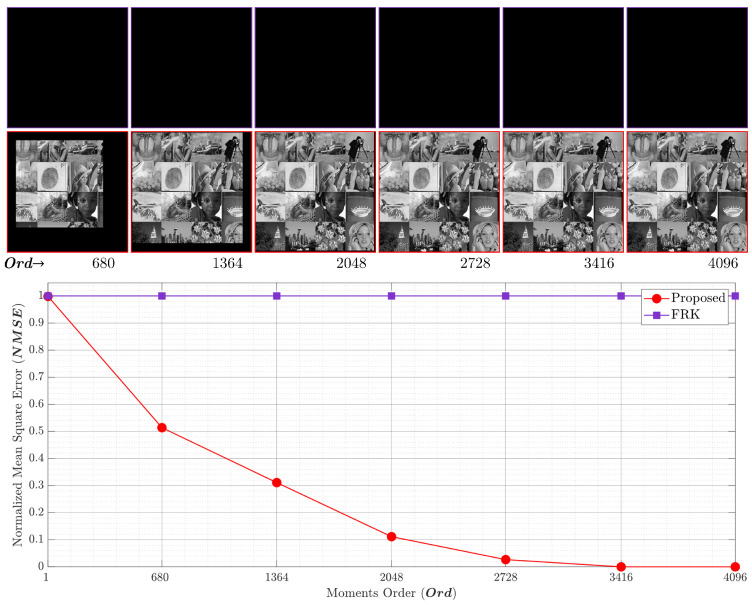
The NMSE performance comparing the proposed algorithm and the RAK algorithm [44] with p=0.4.

Figure 18 shows a new performance evaluation using p=0.4. The results show that the proposed algorithm has the ability to accurately generate the KP coefficients while for the FRK, the KP coefficients remain inaccurate. This is attributed to the zero initial value obtained in the FRK algorithm. It is also worth noting that the proposed algorithm is able to generate KP coefficients for large polynomial sizes and at a high polynomial order, which was experimentally found to be greater than 8192.

Finally, this paper provides a comparison of a maximum polynomial size between the proposed and existing algorithms. The maximum size is obtained for different values of the parameter *p*. For each recurrence algorithm, the polynomial (*R*) is computed first with a size and order of N×N. Then, the following criterion is applied:

(36) $$Err=sum(diagonal\left(R\times R^{T}\right)-I\left(N\right))<Th,$$

where Th is the threshold value over which $10^{-5}$ is chosen. I(N) is the identity matrix with a size of N×N. It is worth noting that the maximum value of *N* is set to 20,480. Table 1 lists the obtained maximum size for each algorithm for different values of parameter *p*. This table demonstrates that the proposed algorithm is capable of generating an orthogonal polynomial with a large size and for different values of parameter *p*. The proposed algorithm outperforms the existing recurrence algorithms for all values of the parameters *p* considered. For example, at p=0.05, the proposed algorithm generates a size of 82× larger than the RAN algorithm, 243× larger than the RAX algorithm, and 16× larger than the FRK algorithm. Thus, the proposed recurrence algorithm can be used to process large signals sizes quickly and accurately.

### 4.3. Computation of the Cost Analysis

The computation cost is considered an important factor to evaluate the performance of the proposed recurrence algorithm [44]. Thus, the computation cost of the proposed algorithm is compared with the existing works. These algorithms the are recurrence algorithm in the *x* direction (RAX), the recurrence algorithm in the *n* direction (RAN), and FRK. The computation cost is performed using the number of computed coefficients. Table 2 shows the ratio of computed coefficients (RCCs) using the proposed algorithm for different polynomials’ sizes. Full moments’ orders are considered. From Table 2, it can be observed that the RCC for small values of *p* and 1−p achieves a low percentage. This percentage increases as the value of *p* increases towards 0.5. The average RCC for p=0.05 and p=0.95 is 9.22% while for p=0.5, the average RCC is 20.35%. This is because the effective coefficient is shaping a circle for p=0.5, and they are shaping a rotated ellipse as the parameter *p* deviates towards 0 or 1, which allows the number of effective coefficients to be reduced. Consequently, the percentage of the computed coefficients is reduced.

Table 3 demonstrates the performance improvement ratio between the proposed and the existing algorithms, namely RAN, RAX, and FRK. The RAN and RAX algorithms compute 50% of the KPCs for all values of parameter *p* because the nx plane is divided into two portions. On the other hand, the FRK algorithm computes 25% of the KPCs as it divides the nx plane into four partitions. The improvement ratio between the proposed and existing algorithms is obtained as

(37) $$\mathrm{Improvement}\;\mathrm{Ratio}=\left(1-\frac{\mathrm{RCC}\;\mathrm{of}\;\mathrm{the}\;\mathrm{proposed}\;\mathrm{algorithm}}{\mathrm{RCC}\;\mathrm{of}\;\mathrm{the}\;\mathrm{existing}\;\mathrm{algorithm}}\right).$$

According to Table 3, the proposed polynomial shows an improvement ratio of 18.64% at p=0.5 when compared to the FRK algorithm and 59.32% when compared to the RAN and the RAX algorithm. The improvement ratio increases as the value of the parameter *p* deviates towards 0 and 1. For example, the results show that at parameter p=0.25, the proposed method achieves an improvement ratio of 29.18% compared to the FRK algorithm, and 64.59% compared to other algorithms. In addition, a maximum improvement ratio of 63.11% is achieved by the proposed algorithm when it compares with the FRK algorithm while it is 81.55% when it compares with the RAN and RAX algorithms.

## 5. Conclusions

This paper described that state-of-the-art algorithms suffer from high error and that the implementation of these algorithms is limited to a specific value of parameter *p*. In addition to this, the state-of-the-art algorithms do not provide any computation of the initial value. Furthermore, to date, no algorithm is proposed for computing the KP coefficients with high-order polynomial and large polynomial sizes. To address these limitations, a new recurrence relation was proposed in this paper. To this end, a new initial value was presented and derived. In addition to this, a new diagonal recurrence relation was introduced. The proposed algorithm divided the KP plane into four triangles and only the coefficients in the upper triangle are computed. The coefficients in the upper triangle were divided into fundamental initial sets, initial sets, Part-1 and Part-2, respectively. The *n*-recurrence, *x*-recurrence, backwards *x*-recurrence, and diagonal recurrence relations were used to compute the values of the coefficients in the upper triangle. In addition, the identities were used to compute the values of the coefficients in the other triangles. The proposed algorithm was evaluated and compared with the existing recurrence algorithms. The comparison was carried out based on the reconstruction error, energy compaction, and computation cost. The experimental results showed that the proposed algorithm achieves a remarkable improvement over the existing algorithms in terms of the maximum size generated and the number of coefficients computed. Although the proposed algorithm outperforms state-of-the-art algorithms, the computational complexity is still high and can be further reduced. This can be achieved by implementing the proposed algorithm in a multi-processing environment (parallelizing) rather than in sequential form, as considered in this paper. Our future work is also directed towards implementing the proposed algorithm for KP with other orthogonal polynomials. This is expected to result in a new OP that has the potential of using orthogonal polynomials as well as the properties of KP coefficients.

## Figures and Tables

**Table 1:** Maximum size of KP that is generated using the proposed and existing algorithms.

*p*	Algorithm				*p*	Algorithm			
	RAN	RAX	FRK	Proposed		RAN	RAX	FRK	Proposed
0.05	248	84	1236	**20,480**	0.55	926	932	2428	**20,480**
0.10	324	132	2250	**20,480**	0.60	808	812	2880	**20,480**
0.15	392	196	2252	**20,480**	0.65	706	708	3368	**20,480**
0.20	462	276	2980	**20,480**	0.70	618	618	3058	**20,480**
0.25	538	436	3400	**20,480**	0.75	538	490	3400	**20,480**
0.30	618	676	3058	**20,480**	0.80	462	318	2980	**20,480**
0.35	710	1234	3368	**20,480**	0.85	390	202	2252	**20,480**
0.40	814	1428	2880	**20,480**	0.90	322	140	2250	**20,480**
0.45	936	1220	2428	**20,480**	0.95	240	88	1236	**20,480**

**Table 2:** The ratio comparison of the computed coefficients of the proposed recurrence algorithm.

		Krawtchouk Parameter (*p*)									
		0.05, 0.95	0.1, 0.9	0.15, 0.85	0.2, 0.8	0.25, 0.75	0.3, 0.7	0.35, 0.65	0.4, 0.6	0.45, 0.55	0.5
Polynomial size (*N*)	1024	10.03	13.31	15.56	17.24	18.52	19.51	20.24	20.75	21.05	21.16
	2048	9.48	12.74	14.99	16.68	17.97	18.96	19.69	20.20	20.50	20.60
	3072	9.25	12.51	14.76	16.45	17.74	18.73	19.47	19.97	20.27	20.38
	4096	9.13	12.38	14.63	16.32	17.61	18.60	19.34	19.85	20.14	20.24
	5120	9.05	12.30	14.54	16.23	17.53	18.52	19.25	19.76	20.06	20.16
	6144	8.99	12.24	14.48	16.17	17.47	18.46	19.19	19.70	20.00	20.10
	7168	8.95	12.19	14.44	16.13	17.42	18.41	19.15	19.65	19.95	20.05
	8192	8.91	12.15	14.40	16.09	17.38	18.38	19.11	19.62	19.92	20.02
Average	9.22	12.48	14.73	16.41	17.71	18.70	19.43	19.94	20.24	20.34	

**Table 3:** Comparing the improvement ratio of the computed coefficients of the proposed algorithm and the existing algorithms.

	Krawtchouk Polynomial Parameter (*p*)									
	0.05, 0.95	0.1, 0.9	0.15, 0.85	0.2, 0.8	0.25, 0.75	0.3, 0.7	0.35, 0.65	0.4, 0.6	0.45, 0.55	0.5
Proposed	9.22	12.48	14.73	16.41	17.71	18.70	19.43	19.94	20.24	20.34
FRK	25.00	25.00	25.00	25.00	25.00	25.00	25.00	25.00	25.00	25.00
RAN and RAX	50.00	50.00	50.00	50.00	50.00	50.00	50.00	50.00	50.00	50.00
Improvement over FRK	63.11	50.09	41.10	34.35	29.18	25.22	22.28	20.25	19.05	18.64
Improvement over RAN and RAX	81.55	75.05	70.55	67.18	64.59	62.61	61.14	60.12	59.52	59.32

## Data Availability

All data are available within the manuscript.

## Appendix A. Proof of Equation (10)

This section presents a proof through Equation (10):

(A1) K0p(x0)=N−1NppNp1−p−Np+N−1=N−1NppNp1−p−Np+N−11/2=(N−1)!(x0)!(N−x0−1)!×px0(1−p)N−1(1−p)x01/2=Γ(N)Γ(x0+1)Γ(N−x0)×1−pp−x0(1−p)N−11/2

By taking the exponential and natural log ($e^{\log}$), then (A1) can be simplified to:

(A2) $$\begin{array}{cc} K_{0}^{p}\left(x_{0}\right) & =e^{\log{\left(\frac{\Gamma (N)}{\Gamma \left(x_{0}+1\right)\Gamma \left(N-x_{0}\right)}\times {\left(\frac{1-p}{p}\right)}^{-x_{0}}{\left(1-p\right)}^{N-1}\right)}^{1/2}}\\ & =e^{\frac{1}{2}\log\left(\frac{\Gamma (N)}{\Gamma \left(x_{0}+1\right)\Gamma \left(N-x_{0}\right)}\times {\left(\frac{1-p}{p}\right)}^{-x_{0}}{\left(1-p\right)}^{N-1}\right)}\\ & =e^{\frac{1}{2}\left(\frac{\log\Gamma (N)}{\log\Gamma \left(x_{0}+1\right)\log\Gamma \left(N-x_{0}\right)}\times {\left(\frac{1-p}{p}\right)}^{-x_{0}}{\left(1-p\right)}^{N-1}\right)}\\ & =e^{\frac{1}{2}\left(\log\Gamma \left(N\right)-\log\Gamma \left(x_{0}+1\right)-\log\Gamma \left(N-x_{0}\right)-x_{0}\log\left(\frac{1-p}{p}\right)+\left(N-1\right)\log\left(1-p\right)\right)}. \end{array}$$
